# An Efficient and Robust Unsupervised Anomaly Detection Method Using Ensemble Random Projection in Surveillance Videos

**DOI:** 10.3390/s19194145

**Published:** 2019-09-24

**Authors:** Jingtao Hu, En Zhu, Siqi Wang, Xinwang Liu, Xifeng Guo, Jianping Yin

**Affiliations:** 1 School of Computer, National University of Defense Technology, Changsha 410073, China; hujingtao17@nudt.edu.cn (J.H.); xinwangliu@nudt.edu.cn (X.L.); guoxifeng1990@163.com (X.G.); 2 School of Cyberspace Science, Dongguan University of Technology, Dongguan 523808, China; jpyin@dgut.edu.cn

**Keywords:** video anomaly detection, unsupervised ensemble learning, random projection, surveillance camera

## Abstract

Video anomaly detection is widely applied in modern society, which is achieved by sensors such as surveillance cameras. This paper learns anomalies by exploiting videos under the fully unsupervised setting. To avoid massive computation caused by back-prorogation in existing methods, we propose a novel efficient three-stage unsupervised anomaly detection method. In the first stage, we adopt random projection instead of autoencoder or its variants in previous works. Then we formulate the optimization goal as a least-square regression problem which has a closed-form solution, leading to less computational cost. The discriminative reconstruction losses of normal and abnormal events encourage us to roughly estimate normality that can be further sifted in the second stage with one-class support vector machine. In the third stage, to eliminate the instability caused by random parameter initializations, ensemble technology is performed to combine multiple anomaly detectors’ scores. To the best of our knowledge, it is the first time that unsupervised ensemble technology is introduced to video anomaly detection research. As demonstrated by the experimental results on several video anomaly detection benchmark datasets, our algorithm robustly surpasses the recent unsupervised methods and performs even better than some supervised approaches. In addition, we achieve comparable performance contrast with the state-of-the-art unsupervised method with much less running time, indicating the effectiveness, efficiency, and robustness of our proposed approach.

## 1. Introduction

Surveillance video is a type of big data that is collected by various sensors in public places. In modern intelligent video surveillance systems, anomaly detection plays an essential role which not only significantly increases monitoring efficiency especially in security area but also alleviates the working burden of traditional video surveillance system operators. Video anomaly detection (VAD) is a hard computer vision task mainly for two specific reasons: (I) The scarcity of abnormal samples. Anomalies are unexpected and infrequent, which make them difficult or even impossible to gather in real-life scenarios. (II) The abstraction of anomaly definition. It is hard to precisely discriminate normal events and abnormal events because the definition of anomaly depends on global context. The same thing can be regarded as a normal or abnormal event in different situations, e.g., a man chasing other man in the bank is considered to be an abnormal event whereas it is a normal event in the playground.

Massive amounts of research have been conducted on video anomaly detection [1,2,3,4,5,6,7,8,9,10,11,12,13,14,15,16,17,18,19,20,21,22,23,24,25]. Depending on whether the labels are available or not, existing anomaly detection techniques in surveillance videos can be categorized as the following three strategies: The first one is the *supervised* setting requires both normal and abnormal samples. Typical approaches in such case are to build predictive models for both normal events and abnormal events, and judge which model fits the input data. However, it is impractical because abnormal events are unexpectable and unbounded in real world, and it is almost infeasible to collect all kinds of abnormal events. Moreover, supervised methods can only be applied on the specific scenes because they are using underlying data and prior knowledge to design limited distributions. Hence researchers pay more attention to the *semi-supervised* setting, which only needs normal behavior to train the normal models. Any input data extremely deviating from normal models is determined as anomaly. Although such techniques can observe unseen abnormal events, it is difficult to obtain a training dataset which covers every possible normal behavior that can occur in real life [26]. The last category is *unsupervised* setting where completely no labels are used in the “training” phase. This kind of methods detects anomalies usually by the properties and characteristics of data themselves. There are quite a few attempts experimenting with unsupervised settings for video anomaly detection. Recently, Del Giorno et al. [1] and Tudor Ionescu et al. [2] tackle the problem with a local change detection method. However, these methods disregard the global information and still have a large gap to be improved. To address these issues, our previous work proposes a two-stage unsupervised video anomaly detection method which uses an autoencoder-based framework [3].

Although existing approaches such as autoencoder-based techniques achieve satisfactory performance in some applications, their nature manners of backpropagation (BP) algorithms lead to massive computational cost in practice, especially for large-scale and high-dimensional video clips. Considering the slow learning speed and huge computation cost in former methods, we propose an efficient ensemble random projection method termed “Ensemble Random Projection-based Reconstruction Loss Neural Network” (E-RR-Net). Our method contains three stages: The first stage, named normalcy estimation, is to roughly estimate normal patterns on contaminated datasets. We adopt a single-hidden-layer feedforward neural network (SFNN), which substitutes the autoencoder framework in existing methods, to reconstruct the input video samples at the output layer through minimizing the reconstruction errors. This is based on the assumption that normal events have smaller reconstruction errors whereas abnormal events’ reconstruction errors are high as demonstrated in Section 3. The essence of first stage technique is that the input layer uses random projection to compress hidden layer into a low-dimensional space, which can act like a bottleneck filtering out redundant information, i.e., abnormal features. The output layer’s weights can be analytically determined by least-square that do not need to be tuned. By doing so, we can reduce huge computation cost that autoencoder has confronted with, meanwhile achieve better performance with less human intervention of tuning numerous parameters. In the second stage, we use One-class Support Vector Machine (OC-SVM) to precisely refine the boundary of normal events’ models evaluated in the first stage. Due to the intrinsic of the random seeds used in random projection, the results need to be further improved more stably and reliably with different initializing settings. So, we introduce the third stage to ensemble anomaly scores of each classifier to enhance the robustness and produce overall judgments. The classifier makes the final decision (normal or abnormal) in an ensemble manner. Compared with the original random projection algorithm, the proposed ensemble technology not only helps to decrease the variance among different nets and enhances the classification performance, but also reduce the number of misclassified samples.

In addition, our proposed method is experimented on three mainstream video anomaly detection benchmark datasets (UCSD Ped1, Ped2 and Avenue). Our method achieves remarkable improvements to anomaly detection performance in contrast to the recent unsupervised methods [1,2] and even surpasses some existing supervised approaches. Moreover, the experimental results also demonstrate the promotion of reducing huge computational cost in comparison with the state-of-the art unsupervised anomaly detection method [3].

Our contribution of this paper can be summarized as follows,

1. To detect anomaly events in videos via a fully *unsupervised* manner, which can avoid cost on labeling specific training data, we proposed a novel three-stage framework called E-RR-Net.
2. We randomly project original video clip features into low-dimensional representations and formulate the normalcy estimation stage as a regression problem, which has a closed-form solution and spends less time in comparison with previous approaches.
3. We introduce ensemble learning to avoid misclassified samples caused by randomness in normalcy estimation stage. We perform multiple independent random projection processing instead of a single one to improve its robustness and effectiveness. *To the best of our knowledge, it is the first time that unsupervised ensemble technology is introduced in video anomaly detection tasks.*

The rest of this paper is organized as follows: The related literature concerning video anomaly detection is reviewed in Section 2, including supervised, semi-supervised, unsupervised and ensemble methods. Section 3 presents the detail of the proposed approach and the description of how the algorithm works. Section 4 shows the experimental datasets and illustrates the data preparation, evaluation metrics, results and discussion of experiments. Section 5 concludes the proposed method.

## 2. Related Work

The issue of anomaly detection in surveillance videos has been extensively studied in the literature. In this section, we summarize the existing research into three categories:supervised, semi-supervised and unsupervised methods. In addition, we introduce ensemble learning technique’s status quo used in video anomaly detection.

### 2.1. Supervised Methods

Most of early research comprises supervised methods which request both normal and abnormal labeled training clips. These methods mainly focus on specific kinds of abnormal events. For example, Chung et al. [4] applied activity recognition in an application targeting nursing home by using Hierarchical Context Hidden Markov Model (HC-HMM). Another work [5] proposed to detect the behavior of human’s falling based on shape variation. Tirunagari et al. [6] focused on face recognition system to prevent face spoofing. In [7], a model was explored for intrusion detection system to protect systems in the Internet age.

Supervised methods are poorly generalized because they do not have the capability to discover the unseen abnormal types of events. It necessarily requires plentiful abnormal events, which are difficult to collect and cause cost-intensively labeling. Moreover, it is difficult to obtain a training data set which covers every possible anomalous behavior that can occur in reality. To overcome these aforementioned issues, more attention has been put on semi-supervised and unsupervised settings.

### 2.2. Semi-Supervised Methods

Semi-supervised approaches are more widely applicable than supervised ones since they just need normal video clips to train models and do not require extra labels for anomaly samples. From the aspect of feature extraction, early works usually used various high-level features to represent the normal behavior [8,9,10], which are easily failed when encountering with complex or crowded scenes with lots of shadows and blurs. To address these problems, most works design various ways of extracting low-level features to better represent the normal behavior. For example, some scholars apply dynamic texture blending theory to detect and localize abnormal behaviors in videos. Mahadevan et al. [14] modeled crowd behavior and recognizes violent scenes using mixture of dynamic texture (MDT). To improve the performance, Li et al. [15] modeled crowded scenes using hierarchical-MDT variations with CRF filters (H-MDT CRF). In addition, Adam et al. [11] proposed a real-time approach using multiple location monitors (MLM) to collect low-level features for video anomaly detection. Kim et al. [12] used a space-time Markov Random Fields (MRF) to model the videos and employs the mixture of probabilistic principal component analysis (MPPCA) to detect abnormal events. The work in [13] proposed to measure normal scores in a non-parametric way by scene dynamics and appearance. Besides these statistic models, sparse representation with dictionary learning is another popular approach for learning regular patterns, which employs sparse coding to build a dictionary for normal events and detects anomalies as the ones with high reconstruction error. Cong et al. [16] adopted multi-scale histogram of optical flow (MHOF) features to learn a normal dictionary and via sparse reconstruction cost (SRC) over the dictionary to detect anomalies. To avoid optimizing the sparse coefficients resulting in consuming lots of time, Lu et al. [17] proposed a sparse combination learning framework without sparse coefficients to build the normal behavior dictionary. In [18], Sun learnt generative dictionaries based on the latent space of variational autoencoder (VAE).

Moreover, many deep learning approaches have been proposed to better capture the representation of normal events under semi-supervised settings.In the work [19], Hasan et al. proposed to use an end-to-end deep learning framework to model normal events, called 3D convolutional autoencoder (Conv-AE). In another work [20], Tran et al. proposed to use a deep spatial sparsity winner-take-all convolutional autoencoder (Conv-WTA) to learn a motion feature representation for anomaly detection. Moreover, Zhao et al. [21] built a novel model called spatio-temporal autoencoder (STAE), which extracted features from both spatial and temporal dimensions by using 3-dimensional convolutions. Another line of works applies recurrent neural network (RNN) on modeling sequential data. The framework in [22] learnt a temporally coherent sparse coding which can map to a stacked recurrent neural network (TSC-sRNN) framework. In contrast to the above methods, Liu et al. [23] used the difference between a predicted future frame and its ground truth by adopting U-Net and Flownet to detect unexpected scenarios.

### 2.3. Unsupervised Methods

Unsupervised setting in video anomaly detection seems more natural to the actual situations in real world. Because human determine the abnormal events through the whole context but not training with labels, unsupervised methods avoid excessive manual labeling. Only a few pieces of research have been conducted in a unsupervised manner because there is no prior knowledge about both normality and abnormality. In the work of [1], Del Giorno et al. performed change detection to detect anomalies by finding which frame was distinguishable from the previous frames. Their simple discriminative learning method was independent of temporal order of abnormal events by permuting the order of frames. Tudor Ionescu et al. [2] applied unmasking technique into video anomaly detection, which iteratively trains a binary classifier to distinguish between two successive video clips while removing the most discriminant features at each loop. If two consecutive events are normal, only small number of features can distinguish them efficiently and the accuracy of classifier will go down severely when eliminating these features. Otherwise, the classification accuracy should stay high even after excluding a certain amount of discriminating features.

However, these two works are all based on local change detection which ignores the global information. To solve this issue, our previous work in [3] proposed a two-stage method: in the first stage, an autoencoder was iteratively trained and adopts a self-adaptive reconstruction loss thresholding scheme to estimate normal events and update autoencoder. Then the second stage introduced one-class SVM to obtain a refined normality model.

Although many efforts have been devoted to unsupervised video anomaly detection, the existing methods suffered from huge space and time consumption and lots of parameters need to be tuned during optimization procedure. In the next section, we introduce our E-RR-Net to overcome these limitations.

### 2.4. Ensemble Learning

A few previous works have embedded ensemble learning into video anomaly detection aiming to improve performance and robustness [27,28,29,30]. In [27,28,29], various sources representative features or descriptors of samples were fused together and fed into a single anomaly detector. The work in [30] used classifier fusion to combine the classification results obtained from two different anomaly detectors.

However, all these prior ensemble video anomaly detection algorithms suffer from the following drawbacks which limit their applications under our scenarios: (i) *most of them only fuse multi-view features into consensus feature representation while little work cares about how to combine various anomaly scores’ information in multiple classifiers and the normalcy level information is missed;* (ii) *the majority of typical ensemble approaches are under the supervised setting that the desired types of anomaly behaviors are given in advance.*

## 3. Proposed Method

**Figure 1:**
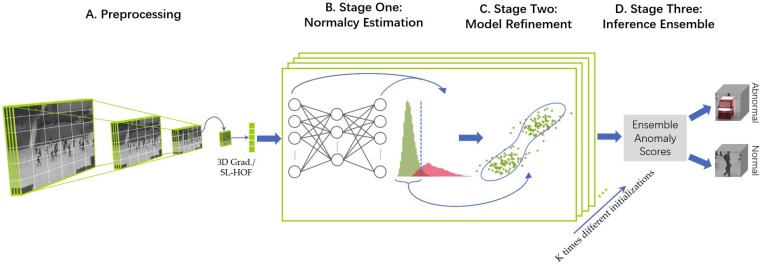
The workflow of our proposed method: (**A**) Preprocessing: Rescale and partition unlabeled videos into spatio-temporal cubes and extract features from them. (**B**) Normalcy Estimation: Roughly evaluate normality from all spatio-temporal cubes without any prior information. (**C**) Model Refinement: Eliminate remaining abnormality and build a model of normalcy. (**D**) Inference Ensemble: Infer the anomaly scores of all video events through the normalcy model under different settings and ensemble the scores.

In this paper, we propose a novel three-stage unsupervised approach to detect abnormal events in videos. After extracting the spatio-temporal feature cubes for preprocessing videos, we employ a simple SFNN to maximally reconstruct the feed-in feature cuboids in the *normalcy estimation* stage. More specifically, the input layer is randomly embedded to the bottleneck hidden layer, which can be fulfilled with fast learning speed. We formulate the optimization goal as a least-square regression problem that can be solved by a closed-form solution and does not need iterative weight tuning. By observing the discriminative reconstruction errors, the frames with low reconstruction errors are roughly estimated as normality. Then, *model refinement* stage adopts a one-class SVM to further exclude abnormality from the first stage. Therefore, we obtain a normality model which can infer the abnormal events. Furthermore, to avoid the sensibility to noise of neural networks’ nature and strengthen the robustness of our proposed method, we construct the *inference ensemble* stage to ensemble the anomaly scores produced by multiple classifiers. Please note that this is the first attempt to use ensemble framework into unsupervised video anomaly detection task. The overall framework is summarized in Figure 1.

### 3.1. Normalcy Estimation Stage

#### 3.1.1. Motivation

**Figure 2:**
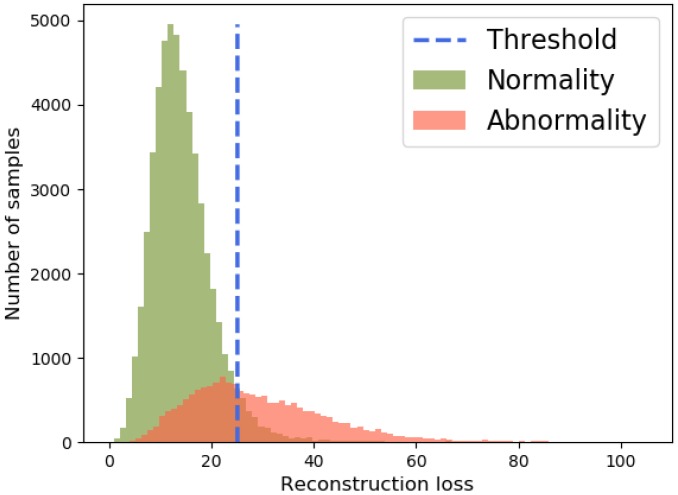
An example of reconstruction discriminative distribution of UCSD Ped2 Dataset.

Followed by [3,19,31,32,33,34], we leverage discriminative reconstruction losses between abnormal and normal events to discover anomaly. So, the definition of abnormality in our work is that abnormal events have relatively high reconstruction errors in contrast with normal events. In other words, the reconstruction losses indicate the probabilities of normal or abnormal events. An experiment is conducted on UCSD Ped2 dataset reconstructing the features of both abnormal and normal events to illustrate our idea. As shown in Figure 2, the distribution of normality reconstruction losses is sharp and gathers on the left of the threshold which demonstrates the low reconstruction losses. On the other side, the average reconstruction losses of abnormality are relatively higher than normality and the abnormality reconstruction losses have wider range on distribution.

This phenomenon comes from the instinct of neural network on reconstruction and the characteristic of imbalanced data. The normality has not only a dominant portion in number but also similar semantic concept, while abnormality is infrequent and discrete. This motivates us to classify the anomalies with their respective construction errors.

Another issue in reconstructing errors which needs to be solved is the heavy computational cost caused by the backpropagation (BP) training procedure. We tackle this problem by designing a SFNN network that using random projection in hidden-layer and try to reconstruct input data as much as possible. The random projection avoids unbearable iterative weight tuning and complicated optimization process in videos. On the other hand, the distance information of data can be well preserved after random projection, which is stated by the Johnson–Lindenstrauss lemma [35]. Moreover, the bottleneck layer can reduce the input features’ dimension and embed them into low-rank representations, which are in fact more representative than the original ones on high-dimensional space. Rather surprisingly, such a simple network can yield comparable or even better performance with a tremendous reduction on execution time. The fast processing speed is essential for big data such as videos. We refer to this stage as RR-Net, which stands for Single Random Projection-based Reconstruction Loss Neural Network.

After collecting all the reconstruction losses of video events, we adopt adaptive labeling to find the optimal threshold in a self-adaptive way. Once the threshold is obtained, the events whose reconstruction losses are under the threshold are classified as normality and vice versa. We find the optimal value of threshold by maximizing the inter-class variance of both normal and abnormal classes. In double peak case, maximizing the inter-class variance is a classical method to discover the threshold adaptively, which can minimize the error probability of wrong classes.

#### 3.1.2. The Algorithm of RR-Net

In the first stage, the input data $X_{n\times d}=\left[x_{1},x_{2},\ldots ,x_{n}\right]$, which is individual feature vector with a dimension of *d*, is compressed into low-dimensional representation in hidden layer $R_{n\times L}$ using random projection. The core idea behind random projection is given in the Johnson–Lindenstrauss lemma [35], which states that if points in a vector space are of sufficiently high dimension, then they may be projected into a suitable lower-dimensional space in a way which approximately preserves the distances between the points. It is worth noting that the new feature space dimension *L* is much less than original feature dimension and is designed to be $O(\sqrt{n})$.

**Figure 3:**
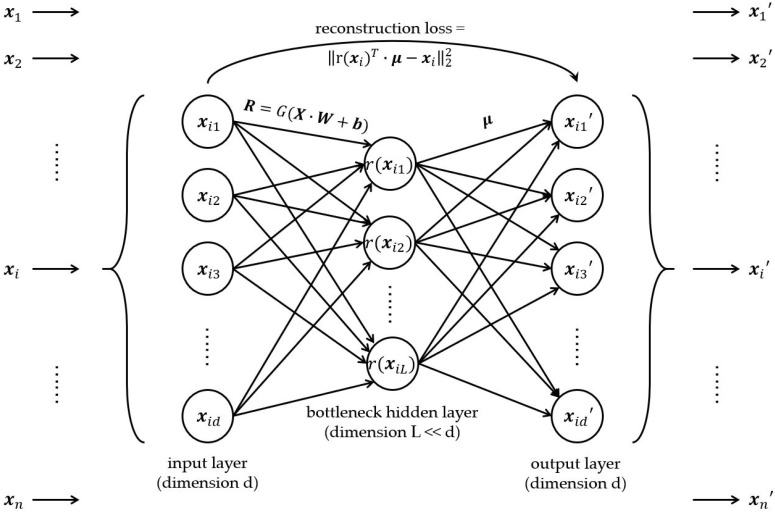
The schematic diagram of RR-Net structure.

By taking random projection matrix $W_{d\times L}$ and random bias $b_{n\times L}$, the output vector of the hidden layer is:

(1) $$R=G\left(X\cdot W+b\right)=[r{\left(x_{1}\right)}^{T},r{\left(x_{2}\right)}^{T},\ldots ,r{\left(x_{n}\right)}^{T}]$$

where G(·) is the activation function. In particular, RR-Net aims to reconstruct input data as close as possible, which can be measured through minimum least-square. According to Bartlett’s theory [36], the smaller norm of weights is, the better generalization performance of networks tends to have. Therefore, the optimization objective function of RR-Net is:

(2) $$\underset{\mu }{min}\;\;\;{∥\mu ∥}_{2}^{2}+\frac{C}{2}\sum_{i}{∥r{\left(x_{i}\right)}^{T}\cdot \mu -x_{i}∥}_{2}^{2}$$

where $\mu_{L\times n}$ is the output weight matrix and *C* is the regularization coefficient that controls the generalization performance. This optimization problem has a closed-form solution determined by Moore–Penrose pseudo inverse [37,38,39]:

(3) $$\mu =R^{T}{\left(\frac{I}{C}+RR^{T}\right)}^{-1}X$$

where I is an identity matrix. Figure 3 displays the structure of RR-Net.

As shown above, RR-Net makes our approach easier for building model and extremely faster to dispose of volume video data. More importantly, RR-Net is fed with the whole video features which has larger perspective and takes the global information into account. The whole algorithm is listed in Algorithm 1.

**Algorithm 1** RR-Net
**Input**: samples $\left[x_{1},x_{2},\ldots ,x_{n}\right]$, *C*.
**Output**: the trained network and the reconstruction losses $\epsilon_{1},\epsilon_{2},\ldots \epsilon_{n}$.
1:Initialize W and b. 2:Update μ by calculating Equation (3). 3:Calculate reconstruction errors with $\epsilon_{i}={∥r{\left(x_{i}\right)}^{T}\cdot \mu -x_{i}∥}_{2}^{2}$.


#### 3.1.3. Adaptive Labeling for Reconstruction Loss

After gathering the reconstruction losses of all video events in Section 3.1.2, next we need to set an adaptive threshold under which the anomaly scores of patterns are classified as normality.

To distinguish the normal and abnormal samples, we find out the optimal value of threshold by maximizing the inter-class variance of both normal and abnormal classes. We formulate the optimal threshold as the solution of the following optimization problem,

(4) $$T_{opt}=\underset{T}{\arg\max}\;p_{normal}\cdot {\left(\epsilon_{normal}-\epsilon \right)}^{2}+p_{abnormal}\cdot {\left(\epsilon_{abnormal}-\epsilon \right)}^{2}$$

where $T_{opt},\epsilon_{normal},\epsilon_{abnormal},\epsilon$ denote the optimal threshold value, the mean of normal samples’ reconstruction errors, the mean of abnormal samples’ reconstruction errors and the mean of all samples’ reconstruction errors respectively. In addition, $p_{normal},p_{abnormal}$ denote the proportion of normal and abnormal samples separately.

Equation (4) can be efficiently solved by enumerating research cross $\left[\epsilon_{min},\epsilon_{max}\right]$. To be specific, the $T_{opt}$ is chosen from *n* values ($\epsilon_{1},\epsilon_{2},\ldots \epsilon_{n}$). When a particular value ($\epsilon_{i}$) is chosen, the value of Equation (4) is calculated and saved as $T_{i}$. When all the $T_{1},T_{2},\ldots ,T_{n}$ are obtained, we clearly have that

(5) $$T_{opt}=\max T_{i},\;i=1,2,\ldots ,n.$$

After $T_{opt}$ is searched by Equation (5), we could label all the samples in the first stage as,

(6) $$Y_{i}=\left\{\begin{array}{cc} \mathrm{abnormal}, & \mathrm{if}\;\epsilon_{i}\geq T_{opt};\\ \mathrm{normal}, & \mathrm{if}\;\epsilon_{i}<T_{opt}. \end{array}\right.$$

Please note that although the first stage could label all the samples, there are still some mislabeled data. This is because there is overlap area at the left of the threshold, which is visualized in Figure 2. To further improve the effectiveness of our model, we design a second stage termed “Model Refinement Stage” to build a tight-boundary one-class SVM classifier introduced in the next section.

### 3.2. Model Refinement Stage

**Figure 4:**
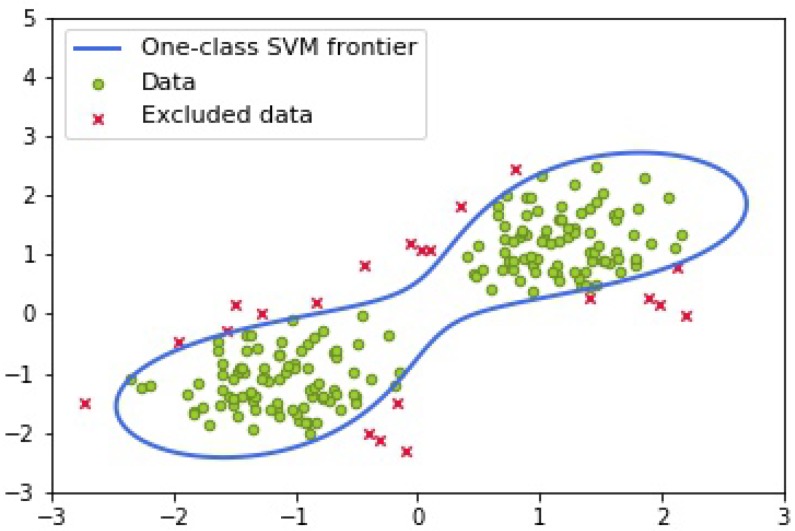
An example of one-class SVM building a tight boundary on a synthetic data with a peanut shape distribution.

After labeling frames based on reconstruction errors in the normalcy estimation stage, most of the anomalies are excluded while there are still a few abnormal samples are mislabeled. To organize a tight boundary to detect abnormal data, we come up with a second model refinement stage which can classify samples more precisely. Inspired by the one-class SVM which is proved to construct a tighter (or narrower) boundary gap [40], we adopt the one-class SVM to leave out the small percentage of samples who are mislabeled as normality in the first stage. To make more explicit explanation for its working principle, we visualize the tight frontier of one-class SVM on a peanut shape dataset in Figure 4.

In this stage, we first consider features from those spatio-temporal cubes at the same spatial location as samples that need to be modeled by one-class SVM, disregarding the temporal relations among cubes. Formally, given the *s* samples $\left\{x_{1},x_{2},\ldots ,x_{s}\right\}$ that need refinement and a feature mapping function ϕ(·) that transfers data to a high-dimensional space H, one-class SVM is desired to seek a separable hyper-plane Ω: $z^{T}\cdot \phi \left(x\right)-\rho =0$, where z is the normal vector of Ω. The hyper-plane Ω can separate the new feature representations $\phi \left(x_{1}\right),\phi \left(x_{2}\right),\ldots ,\phi \left(x_{s}\right)$ from the origin point *O* of H that enables *O* to have the largest margin to itself. The optimization function can be expressed as follows [41],

(7) $$\begin{array}{cc} \min_{z,\xi ,\rho } & \;\frac{1}{2}{\left||z|\right|}^{2}+\frac{1}{\nu s}\sum_{i=1}^{s}\xi_{i}-\rho \\ s.t. & \;z^{T}\cdot \phi \left(x_{i}\right)-\rho +\xi_{i}\geq 0,\;\xi_{i}\geq 0,\;\forall i, \end{array}$$

where $\xi_{i}$ is the slack variable for $x_{i}$, and ν is the regularization parameter. By introducing slack margins, the trained one-class SVM may be able to yield a softer decision boundary excluding those data with large deviations as noises. Equation (7) can be efficiently solved as a quadratic programming problem with existing scikit-learn packages [42]. After doing so, our model can obtain a refined decision boundary that compactly surrounds the given data, while excluding those severely deviated data with $\xi_{i}<0$. At last, any data outside the decision boundary is classified as abnormal events in video clips.

### 3.3. Inference Ensemble Stage

Depending on the random selection of weights and biases for hidden nodes, our network decreases the learning time dramatically compared to previous approaches. However, the parameters are randomly initialized and may contain non-optimum, thus the performance might be unstable. Consequently, we propose to perform multiple independent random projection classifiers instead of a single one to overcome the shortcomings of single random projection detector, then ensemble various anomaly scores as the final abnormality estimation scores.

**Figure 5:**
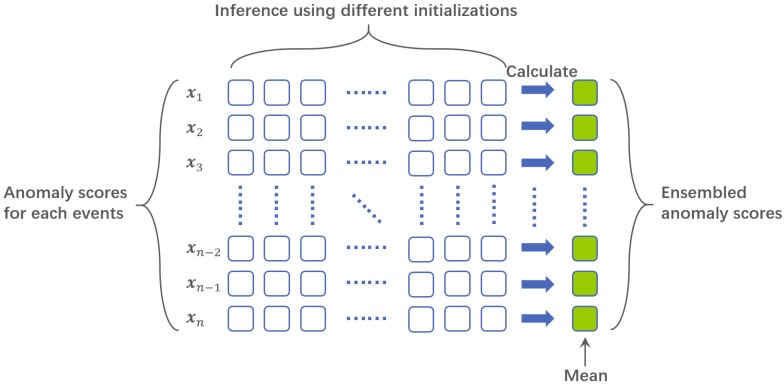
An illustration of our proposed anomaly scores ensemble under different initializations and final anomaly scores computation.

In our paper, to avoid the mislabeling by random parameter initialization, we propose to ensemble the different anomaly scores obtained from multiple classifiers in the partition level. To be specific, each fully trained one-class SVM classifier could rank the given frames’ abnormality. However, due to the initializations of the input layer brought by randomness, some of these rank scores may deteriorate evaluation performance. Under the fully unsupervised setting, lack of prior knowledge makes ensemble learning quite a hard task. We are expected to adopt the final decision scores by the mean of a given set of scores. It is worth noting that to the best of our knowledge, it is the first time that unsupervised ensemble technology is introduced in video anomaly detection which improves robustness and effectiveness. The workflow of our ensemble method is illustrated in Figure 5.

### 3.4. Extensional Discussion and Complexity of Proposed Method

*Discussion:* Compared to our previous work [3], we adopt a quite different way to reconstruct video samples, which efficiently avoid the massive computational cost. To be more specifically, we use random projection to compress data into a new hyperspace and then formulate the optimization goal as a least-square regression problem instead of autoencoder way in [3]. In contrast, the work in [3] applied the backpropagation (BP) based on gradient descent, which makes it quite slow during training process. Moreover, ensemble learning is elaborately added to our method to eliminate the negative effect of randomness caused by random projection. Furthermore, our ensemble framework can be conducted under parallel programming that do not increasing processing time. By doing so, our method achieves comparable or even better performance compared to [3] with much less running time, which is remarkable for the big data in video form under the unsupervised setting.

*Parallelization*: The ensemble process can be easily paralleled since each classifier is “trained” and performed independently. Therefore, although multiple anomaly classifiers may lead more computational time than single one, our running time is nearly the same with single detector through parallelization processes.

*Time Complexity*: In this section, we will give theoretical analysis of the time complexity of our proposed ensemble three-stage method. In the first stage, it is a random projection optimization problem with closed-form solution. As for this network, we only need to calculate the weight matrix $\beta_{L\times n}$. The complexity is $O(n^{2}L+L^{3}+Ld)$ [43]. The second stage is an one-class SVM training process whose complexity is $O(s^{3})$ [44], where *s* represents the number of samples who are regarded as normal ones in the first stage.

The time efficiency of our method compared to previous methods is mainly attributed to the first stage deploying random projection. Existing methods adopt gradient-based autoencoder of which the training time is too long to bear. Obviously, our method reduces the heavy time burden and simplifies the optimization procedure. Further experimental results demonstrate the efficiency of our method.

## 4. Evaluation

### 4.1. Datasets

**Figure 6:**
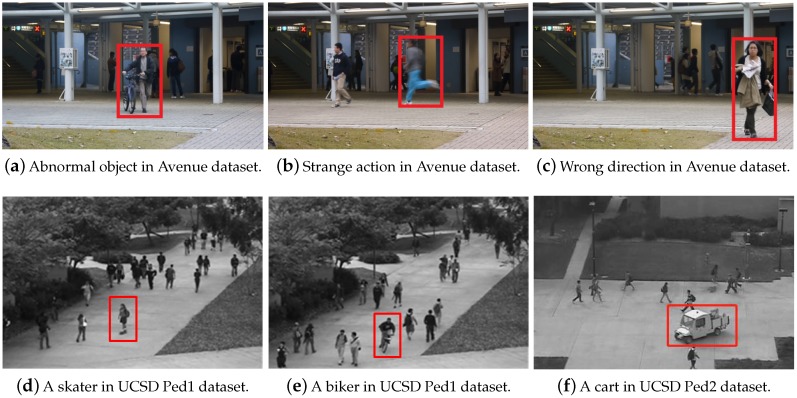
The representative abnormal events in the benchmark datasets (highlighted in red box).

The experiments are conducted on three standard benchmarks for video anomaly detection: UCSD Ped1 and UCSD Ped2 and Avenue dataset. Please note that no label information is pre-given in all these experiments. There is no need to build a normality model only using normal training datum, so both normal and abnormal video events are fed into our model in an unsupervised manner. The details of dataset total number of frames and frame resolution are shown in Table 1. Figure 6 displays the representative abnormal events in three benchmarks.

The prevalent benchmark UCSD dataset (http://www.svcl.ucsd.edu/projects/anomaly/dataset.html) [14] records a video footage of pedestrian walkway collected from the video surveillance from two different places of UCSD campus, i.e., UCSD Pedestrian 1 (Ped1) and UCSD Pedestrian 2 (Ped2). It contains some common anomalies happened on the pavements, such as skaters, carts, bikers and other entering of undesirable objects except pedestrians. The motion of pedestrians in an unexpected area is also considered to be an irregular event. Both UCSD Ped1 and Ped2 are widely used datasets. Usually common practices are evaluated on these two parts separately. Moreover, the UCSD Ped1 dataset contains a relatively high proportion of abnormality, which makes it quite challenging. The UCSD Ped2 dataset has fewer video frames and larger frame resolution than Ped1, which makes it more frequently used. Avenue dataset (http://www.cse.cuhk.edu.hk/leojia/projects/detectabnormal/dataset.html) [17] is another well-labeled and recent benchmark dataset captured by surveillance cameras in CUHK campus’s avenues. To close to the real situation, there are some slight camera shakes in the test video clips so that it focuses on moving objects as anomalies. It contains various type of abnormal events such as running, throwing objects, loitering, pushing bike and so on.

### 4.2. Evaluation Metric

Following the previous research on video anomaly detection [15,17], we use frame-based area under receiver operating characteristics (ROC) curves (AUC) and equal error rate (EER) to quantitative evaluate our performance. We adopt frame-level criteria described in [14]: a video frame is considered to be abnormality as long as method detects at least one pixel is anomalous. Given that the dynamic nature of videos, measuring abnormal events at pixel-level is unwieldy and unnecessary. In common settings, “positive” denotes an abnormal frame and “negative” means a normal frame. The ROC curve represents the variation of true positive rate (TPR) versus false positive rate (FPR) by gradually changing the threshold to discover anomaly. AUC denotes the area under the corresponding ROC curve of which higher value indicates better performance. The ratio of misclassified video frames FPR = 1 − TRP is valued at equal error rate (EER).

### 4.3. Video Preprocessing and Implementation Setup

Given the input videos, we first rescale each video frame to different pixels to detect abnormality with different sizes. As shown in Table 1, different dataset settings are distinguishing. Second, we split the video frames into non-overlapping M×N patches with equal size, and temporally consecutive *D* patches are partitioned into a spatio-temporal cube. A cuboid, which is filtered the background, can be treated as a single video event. As a general practice in video surveillance, we adopt M=10, N=10 and D=5. Next, 3D Gradient features [17] for Avenue dataset and SL-HOF features [45] for UCSD dataset are extracted from the cuboids for better representation.

After data preprocessing, all these features are fed into stage one for normalcy estimation. Empirically, the weights and bias of random projection are generated from a uniform distribution between [-1,1]. The regularization *C* in Equations (2) and (3) is not used. We choose ReLU, which is simple and computationally costless, as the activation function of random projection. In the model refinement stage, we use the Gaussian kernel $\kappa \left(x,x^{\prime }\right)=exp\left(-{\|x-x^{\prime }\|}^{2}/2\sigma^{2}\right)$ for one-class SVM, and the hyper-parameters of it are determined by five-fold cross validation.

All experiments are carried out under Python 3.6 environment running in 64GB RAM and 3.60 GHz Intel i7 7820X processor.

### 4.4. Efficiency

As demonstrated in Table 2, our proposed method has proven incredibly speed up on three datasets for unsupervised video anomaly detection. It is obvious that our method has the advantage on efficiency. The remarkable improvements on efficiency are essential for handling with vast video data in real life. Apparently, the efficiency of our approach is improved several or even ten times compared with the state-of-the-art method [3]. The slow tuning of all the parameters hinders this gradient-based method [3] from meeting the requirement in video tasks.

It should be noted that the processing time cannot be divided into train and testing time respectively in an unsupervised manner. Other than supervised and semi-supervised methods, unsupervised normalcy model learning itself triggers the generation of anomaly scores. To be expedited in the future, it is reasonable to expect that a faster implementation such as C++ will accelerate our approach.

### 4.5. Effectiveness

#### 4.5.1. Results on UCSD Ped1 Dataset

Our method is competitive over two state-of-the-art unsupervised approaches as well as several mainstream supervised methods on UCSD Ped1 dataset. The results reported in Table 3, Table 4 and Table 5 are the best performances of different settings. The comparable results are represented in Table 3 of which the best performers are formatted in boldface. With highly efficient computation, our approach surpasses all the unsupervised methods, surprisingly. In contrast of [2], a gain of AUC up to 12.5% has been made by our method. This is owing to the global information is adopted to calculate the reconstruction losses of all spatio-temporal cuboids. However, the method in [2] adopted local change detection which meet the dilemma confronting with frequent abnormal events. As for [3], the complex autoencoder neural network is very likely to overfit the data with anomalies without any prior information. Random projection presents a natural solution to address this predicament. Moreover, another unsupervised method [1] does not evaluate on UCSD dataset due to the high ratio of abnormal events occurred in video samples.

**Figure 7:**
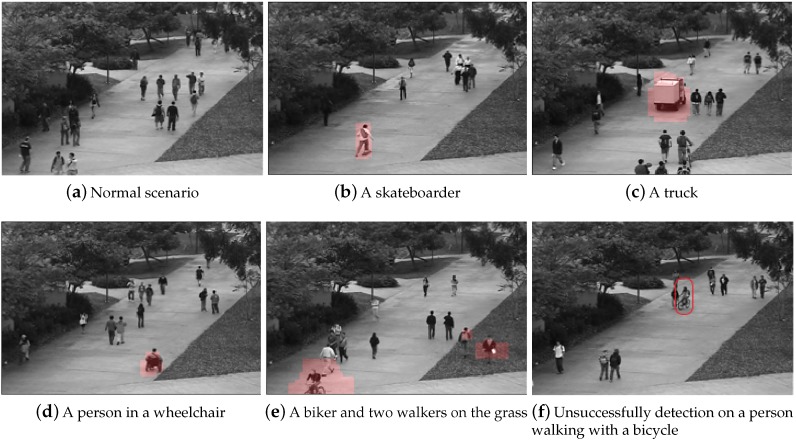
The visualization results of our method on UCSD Ped1 dataset (highlighted in red).

Figure 7a shows the representative normal scene and Figure 7b–e display different abnormal events detected by our method on UCSD Ped1 dataset. The proposed method can successfully discovers a variety of anomalies, which is unexpected on the walkway such as a skateboarder in Figure 7b, a truck in Figure 7c, a person in a wheelchair in Figure 7d, a bicycler and two men walking across the lawn in Figure 7e. It should be pointed out that our method fails to find a person walking with bike displayed in Figure 7f. One possible reason is that we extract SL-HOF features on UCSD dataset, which focus on the movement, and he is walking slowly like a normal pattern.

#### 4.5.2. Results on UCSD Ped2 Dataset

Table 4 reports satisfactory experiment results of our approach for video anomaly detection on UCSD Ped2 dataset. Even though the AUC of our method is merely 0.5% worse than the approach in [3], the proposed method is 13.7% higher than [2]. Meanwhile, our method reaches comparable or better performance than half of supervised methods significantly.

**Figure 8:**
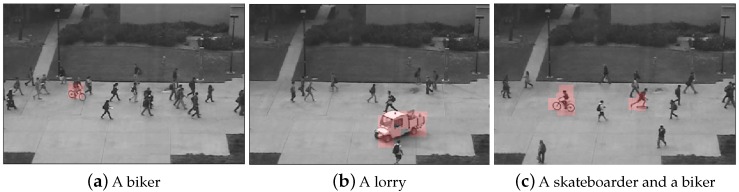
The visualization results of our method on UCSD Ped2 dataset (highlighted in red).

We provide some visualized examples detected by our method on UCSD Ped2 dataset in Figure 8. Our approach effectively discovers various types of abnormal events such as a biker in a crowd (Figure 8a) and a lorry (Figure 8b). It is worth noticing that our method successfully localizes multiple anomalies in one frame shown in Figure 8c.

#### 4.5.3. Results on Avenue Dataset

The quantitative evaluation results indicated by Table 5 confirm the validity of our approach. In most case, our approach outperforms the prevalent unsupervised methods as well as supervised methods. Compared with the unsupervised approaches of Del Girno et al. [1] and Unmasking [2], our method brings an improvement of 5.9% and 3.6% AUC, respectively. In addition, our method reaches about the same level as the state-of-the-art supervised methods and even exceeds it on EER. Overall, our performance on three datasets are remarkable.

**Figure 9:**
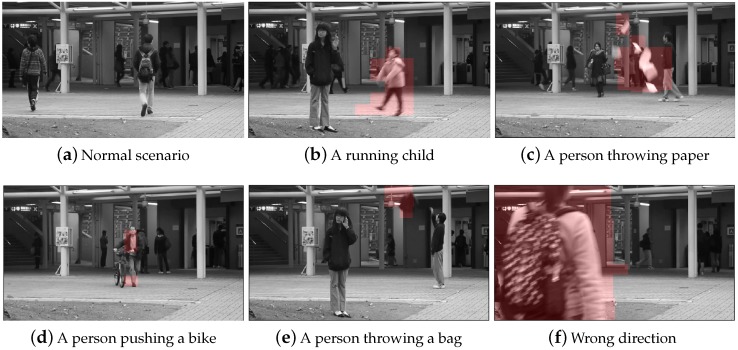
The visualization results of our method detection on Avenue dataset (highlighted in red).

Figure 9 illustrates our fruitful unsupervised anomaly detection results on Avenue dataset. We represent a normal event as an example in Figure 9a. Moreover, we can easily identify different abnormal events, such as a running child in Figure 9b, a person throwing paper in Figure 9c, a person walking with a bike Figure 9d, a person throwing a bag in Figure 9e and a girl walking in wrong direction showed in Figure 9f. As can be seen, our proposed method works well on detecting anomalies that requires no labeled training samples.

### 4.6. Ablation Study

In this section, we conduct experiments to illustrate the effectiveness of combining multiple anomaly detectors’ scores as opposed to single one. The number of ensembles used in the experiments *m* are 1 (referring to single), 3, 5 and 10. Our purpose of ensemble various anomaly scores is to improve the decision system’s robustness and eliminate the randomness of weights and bias matrices which importantly influence the performance. Hence for the three benchmark datasets, we set different random seeds that produce corresponding initializations of weight matrix W and bias matrix b. We repeat each of the four settings (m=1,3,5,10) 10 times, and report their performance in Table 6 and Table 7.

**Figure 10:**
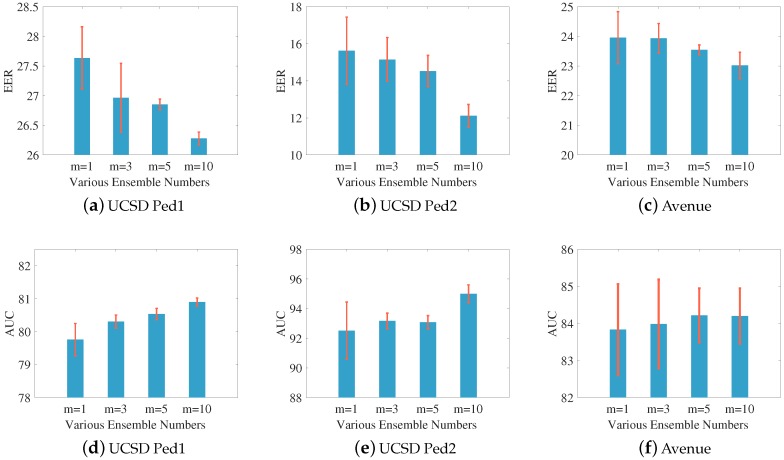
The EER and AUC comparison (mean ± std) on three benchmark datasets under different settings.

We also plot the mean and standard deviation (std) comparison of the EER and AUC in Figure 10. As can be observed, with the increasing of *m* (ensemble components), the whole decision system achieves improved and stable performance. The mean of desired evaluation metrics are enhanced while the variance become much lower, which sufficiently demonstrates the effectiveness and robustness of our proposed ensemble technology.

## 5. Conclusions

In this paper, we develop a novel three-stage framework to tackle the unsupervised anomaly detection task with fast learning speed on videos. The first stage is originally proposed to break through the slow execution dilemma caused by back-prorogation for iterative tuning of the networks’ parameters in previous works. It randomly projects the input data into a low-dimensional hidden space and analytically determines the output weights, which saving tremendous time. Next, we deploy the discriminative reconstruction losses of normality and abnormality for normalcy estimation. By considering the whole context rather than the local change detection, the global information from video context can be fully exploited. In the second stage, we adopt one-class SVM to build a more precise envelope around normal data that estimates remaining abnormal events. Furthermore, an ensemble method is proposed to eliminate the biases brought by random initializations and is desired to achieve robust classification results. Experimental results, which obtain superior performance at a much faster learning speed on three prevalent datasets, show our method’s efficiency, robustness and effectiveness.

In the future, more ensemble technologies can be applied into our framework and exploiting the comparison of different ensemble methods will be an interesting work.

## Figures and Tables

**Table 1:** Details of datasets used in our experiments.

	Frames (Total)	Pixels	Rescaled Pixels
UCSD Ped1	14,000	238 × 158	160 × 240, 120 × 180, 100 × 150
UCSD Ped2	4560	360 × 240	180 × 270, 120 × 180, 100 × 150
Avenue	30,652	640 × 360	120 × 160, 30 × 40

**Table 2:** Runtime comparisons of proposed method and state-of-art unsupervised VAD method.

	UCSD Ped1	UCSD Ped2	Avenue
Proposed	1 h	0.13 h	0.27 h
Wang et al. [3]	6.8 h	1.4 h	2.3 h

**Table 3:** Frame-level EER and AUC evaluations on UCSD Ped1 dataset (“-” denotes that the result is not reported).

Method		Frame-Level	
		EER	AUC
Supervised	Adam et al. [11]	38%	65%
	MPPCA [12]	40%	59%
	SF [46]	31%	67.5%
	SF+MPPCA [14]	32%	77%
	MDT [14]	25%	81.8%
	Bertini et al. [13]	31%	-
	SRC [16]	19%	86%
	Lu et al. [17]	15%	91.8%
	HMDT-CRF [15]	18%	-
	CAE [19]	27.9%	81%
	STAE [21]	15.3%	92.3%
	WTA-CAE [20]	14.8%	91.6%
	Liu et al. [23]	-	83.1%
Unsupervised	Unmasking [2]	-	68.4%
	Wang et al. [3]	29.2%	77.8%
	Proposed	26.3%	80.9%

**Table 4:** Frame-level EER and AUC evaluations on UCSD Ped2 dataset (“-” denotes that the result is not reported).

Method		Frame-Level	
		EER	AUC
Supervised	Adam et al. [11]	42%	63%
	MPPCA [12]	30%	77%
	SF [46]	42%	63%
	SF+MPPCA [14]	36%	71%
	MDT [14]	25%	85%
	Bertini et al. [13]	34%	-
	HMDT-CRF [15]	18.5%	-
	OWC-MTT [47]	13.9%	94.0%
	CAE [19]	21.7%	90%
	STAE [21]	16.7%	91.2%
	TSC-sRNN [22]	-	92.2%
	WTA-CAE [20]	8.9%	96.6%
	Liu et al. [23]	-	95.4%
Unsupervised	Unmasking [2]	-	82.2%
	Wang et al. [3]	8.9%	96.4%
	Proposed	10.5%	95.9%

**Table 5:** Frame-level EER and AUC evaluations on Avenue dataset (“-” denotes that the result is not reported).

Method		Frame-Level	
		EER	AUC
Supervised	Lu et al. [17]	-	80.9%
	CAE [19]	25.1%	70.2%
	STAE [21]	24.4%	80.9%
	TSC-sRNN [22]	-	81.7%
	WTA-CAE [20]	24.2%	82.1%
	Liu et al. [23]	-	84.9%
Unsupervised	Del Giorno et al. [1]	-	78.3%
	Unmasking [2]	-	80.6%
	Wang et al. [3]	23.9%	85.3%
	Proposed	23.0%	84.2%

**Table 6:** Frame-level EER evaluations (mean ± std) with different settings on three benchmark datasets.

	Settings	m=1	m=3	m=5	m=10
Datasets					
UCSD Ped1		27.64±0.53	26.96±0.30	26.85±0.09	26.28±0.10
UCSD Ped2		15.63±1.81	15.15±1.18	14.53±0.85	12.11±0.62
Avenue		23.96±0.88	23.94±0.50	23.54±0.17	23.01±0.44

**Table 7:** Frame-level AUC evaluations (mean ± std) with different settings on three benchmark datasets.

	Settings	m=1	m=3	m=5	m=10
Datasets					
UCSD Ped1		79.76±0.49	80.30±0.20	80.54±0.17	80.90±0.12
UCSD Ped2		92.52±1.93	93.17±0.53	93.08±0.46	94.26±0.60
Avenue		83.83±1.23	83.99±1.20	84.22±0.73	84.21±0.75

## Abbreviations

The following abbreviations are used in this manuscript:

E-RR-Net	Ensemble Random Projection-based Reconstruction Loss Neural Network
RR-Net	Single Random Projection-based Reconstruction Loss Neural Network
SFNN	Single-hidden-Layer Feedforward Neural Network
BP	Backpropagation
SVM	Support Vector Machine
OCSVM	One-class Support Vector Machine
RL	Reconstruction Loss
RP	Random Projection
VAD	Video Anomaly Detection
CUHK	The Chinese University of Hong Kong
UCSD	University of California, San Diego
ReLU	Rectified Linear Unit
ROC	Receiver Operating Characteristics
AUC	Area under ROC Curves
EER	Equal Error Rate
TPR	True Positive Rate
FPR	False Positive Rate
